# Urinary Screening for Detection of Renal Abnormalities in Asymptomatic School Children

**DOI:** 10.5812/numonthly.3528

**Published:** 2012-06-20

**Authors:** Prince Parakh, Nisha K Bhatta, Om P Mishra, Pramod Shrestha, Sunil Budhathoki, Shankar Majhi, Arvind Sinha, Kanchan Dhungel, Rahul Prabhakar, Niladri Haldhar

**Affiliations:** 1 Departments of Pediatrics and Adolescent Medicine 1, B. P. Koirala Institute of Health Sciences (BPKIhS), Dharan, Nepal; 2 Department of Pediatrics, Institute of Medical Sciences, Banaras Hindu University, Varanasi, India; 3 Departments of Biochemistry, B. P. Koirala Institute of health Sciences (BPKIhS), Dharan, Nepal; 4 Departments of Pathology, B. P. Koirala Institute of health Sciences (BPKIhS), Dharan, Nepal; 5 Departments of Radiodiagnosis and Imaging, B. P. Koirala Institute of health Sciences (BPKIhS), Dharan, Nepal

**Keywords:** Urinary Screening, School Children, Renal Diseases

## Abstract

**Background:**

Urinary screening tests for early detection of renal diseases in asymptomatic school children and adolescents are important in the detection of silent renal diseases.

**Objectives:**

The purpose of the study was to determine the prevalence of occult renal diseases by dipstick test (reagent strips) in asymptomatic Nepalese children.

**Patients and Methods:**

A total of 2,243 school children, aged 5–15 years, were screened for urinary abnormalities using dipstick test screening. The children who tested positive in the first screening were re-tested after 2–4 weeks.

**Results:**

In the first screening, 123 children (5.5%) tested positive for isolated hematuria and proteinuria and for combined hematuria and proteinuria. Of these children, 16 (0.71%) cases tested positive in a second screening. Subsequently, 1 child from the secondary screening group was lost to follow up, 5 tested normal and 10 revealed abnormalities. Glomerulonephritis was the most commonly detected disorder (50%).

**Conclusions:**

Urinary screening was found to be useful in identifying occult renal diseases in asymptomatic children. Urinary screening would therefore not only help in early detection but also in the prevention of the deterioration of renal function later in life.

## 1. Background

Early identification and treatment of kidney diseases in children and adolescents are important initial steps in prevention of chronic kidney diseases (CKD). CKD in children may be too covert for early detection and its occurrence is a worldwide health problem (1-3). Many countries face the burden of their own endemic renal conditions, such as IgA nephropathy in Asia, Hepatitis B-related nephropathy in Asia and Africa, and HIV-associated nephropathy in Africa, Asia, Europe, and the UnitedStates (4, 5). The simplest and least expensive method of screening apparently healthy subjects is urinalysis (6, 7). Several studies have used reagent strips and have documented their effectiveness in detecting urinary abnormalities (8, 9).

## 2. Objectives 

Persistent proteinuria has been shown to be associated with CKD. Similarly, hematuria is one of the most common urinary abnormalities seen in children with parenchymal renal diseases (10). There is wide variation in the incidence and pattern of renal diseases in Asia (11-13). However, there are no published studies on early detection of renal conditions in children from Nepal. Therefore, the present study was prospectively conducted as a urinary screening of asymptomatic school children and adolescents.

## 3. Patients and Methods 

This study was performed from January 2010 to June 2011. A total of 2,243 children aged 5–15 years from 5 different schools of the town of Dharan, Nepal were included in the study. Assuming that the prevalence of urinary abnormalities in 5–15-year olds is 7.2% (14), and given the population of 27,140 in this age group in Dharan and 85% as the power of study, the necessary sample size was determined to be 2038. Anticipating 10% of the subjects would be inaccessible or would fail to report to follow-up, a total of 2243 asymptomatic children were screened. Children with pre-existing renal or any other systemic diseases, children on steroid therapy, and children whose parents refused to give consent were excluded. The protocol of the study was approved by the Institute Ethics Committee and informed written consent was obtained from parents and the school administration. The study protocol conforms to the ethical guidelines of the 1975 Declaration of Helsinki.

### 3.1. Screening Protocol

**Figure 1:**
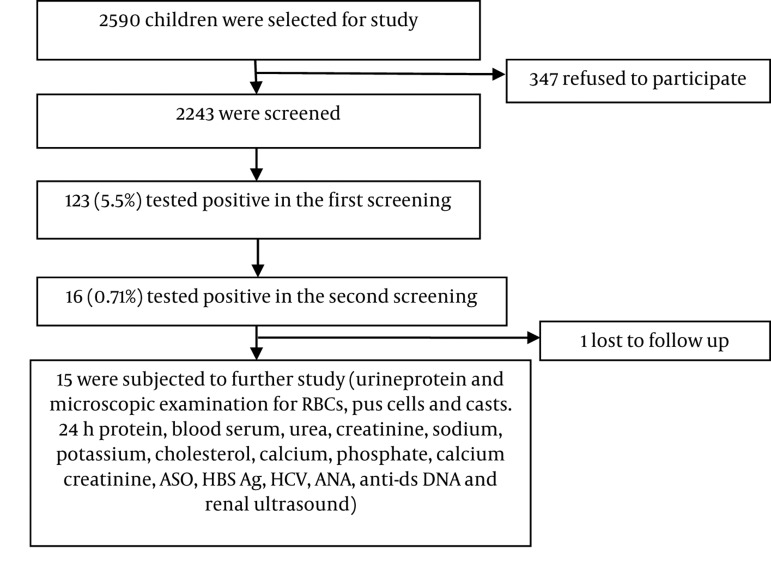
Study Flow and Screening Protocol.

Instructions written in the local language about proper methods of collection of urine samples were sent home to parents in advance. The first morning urine sample was obtained from each child in a clean 50mL vessel, which was tested with a urinary dipstick (Insight Urinalysis Reagent Strips, Acon Laboratories, San Diego, CA, USA) for hematuria and/or proteinuria as a first screening test. The second screening test was performed 2–4 weeks later by urinary dipstick on 123 children who had tested positive in the first screening (Figure 1).

Children with abnormal urinary findings in the second screening were brought to the Department of Pediatrics and tested for urinary microscopic, culture, 24-hr urinary protein, and spot urinary calcium/creatinine ratio. Blood studies included serum urea, creatinine, sodium, potassium, total protein, albumin, cholesterol, calcium, phosphate, anti-streptolysin antibody (ASO) titer, hepatitis B surface antigen (HBsAg), anti-hepatitis C antibody (anti-HCV), anti-nuclear antibody (ANA), and anti-double stranded DNA (anti-dsDNA). Renal ultrasound was performed on all 15 cases. A detailed history was taken, and physical and systemic examinations were performed on all children with urine abnormalities in the second screening. Anthropometric parameters such as weight, height, mid-left upper-arm circumference, and blood pressure were recorded.

### 3.2. Statistical Analysis 

The statistical analysis was performed using SPSS version 16.0. Chi-squared and Student’s t-tests were applied to compare proportions and mean differences, respectively. A *P* value of less than 0.05 was considered significant.

## 4.Results 

Consent forms were given to 2590 asymptomatic school children aged 5–15 years, but only 2243 completed forms were returned. Thus, the first screening urinalysis was performed on 2243 children. There were 1217 males (54.2%) and 1026 females (45.7%). The Mongolian race constituted 44.8% (1006) of the screened population and 55.1% (1237) were non-Mongolian children. The school children were divided into two age groups: 5–10 years and 11–15 years. There were 847 children in the 5–10 year age group and 1396 children in the 11–15 year age group. In the first screening, 123 children (5.5%) were found to test positive for hematuria and/ or proteinuria. The male to female ratio was 0.84:1 in positive children.

Basic parameters such as gender, race, systolic and diastolic blood pressure, weight, height, and mid-upper arm circumference were analyzed, and it was observed that only the mean value of diastolic blood pressure (P = 0.02) was significantly higher in children with positive urinary tests compared to negative cases.

The frequency of positive children in the first and second screenings is presented in Table 1.The proportions of children who tested positive for isolated hematuria (IH), isolated proteinuria (IP), and combined hematuria and proteinuria (CHP) were comparable between the 5–10 and 11–15 age groups. The prevalence of hematuria was higher (3.5%) in the latter age group than in the former (1.54%), while proteinuria was the same between the two groups. Of 123 positive cases found in the first screening, 16 (0.71%) were found to be positive in the second screening. The overall incidence of isolated hematuria, isolated proteinuria, and combined hematuria and proteinuria were 0.40%, 0.22%, and 0.09%, respectively, among the studied population. Of the 16 children who tested positive in the second screening, one was lost to follow-up and the remaining 15 were subject to further study. Subsequently, 5 were found to be normal, and renal abnormalities were detected in 10. The features of these abnormalities are summarized in Table 2. Glomerulonephritis was the most commonly detected abnormality and was found in 5 cases (lupus nephritis in 4 children and acute post-streptococcal glomerulonephritis in 1 child).

**Table 1:** Frequency of Positive Children in First and Second Urinary Screening

**Screening**	**Age, y**	**Actual Number**	**Hematuria, No. of Positive Cases (%)**	***P***	**Proteinuria, No. of Positive Cases (%)**	***P***	**Hematuria, and Proteinuria, No. of Positive Cases (%)**	***P***
First Urinalysis				0.082		0.896		0.279
	5–10	847	13 (1.54)		30 (3.54)		3 (0.35)	
	11–15	1396	37 (3.5)		48 (3.44)		2 (0.14)	
Second urinalysis		123	9 (0.40)^a^		5 (0.22)^a^		2 (0.09)^a^	

^a^Percentages (in second urinalysis) are expressed as a percentage of the total study population (number of positive children ÷ total screened population × 100)

**Table 2:** Renal Abnormalities Detected After Final Workup

**Case**	**Age, y**	**Gender**	**Findings**	**Comments**
1	11	Male	ASO^a^ titer = 400IU/mL	Post streptococcal glomerulonephritis – resolved
2	6	Female	Nephrotic range proteinuria (4.4 gm/1.73m^2^ /24hr), serum albumin 2.3g/ dL and cholesterol 230mg/dL	Responded to prednisolone
3	13	Female	Mild right hydroureteronephrosis on USG^a^	Under regular follow up
4	13	Female	Internal echoes in urinary bladder on USG	Cystitis-resolved
5	15	Male	8 mm calculus in mid pole of right kidney on USG	Under regular follow up
6	12	Male	Bilateral pelviureteral junction obstruction on USG	Surgical intervention
7	8	Male	ANA^a^ and dsDNA^a^ positive	Lupus nephritis
8	10	Male	ANA and dsDNA positive	Lupus nephritis
9	9	Female	ANA and dsDNA positive	Lupus nephritis
10	8	Female	ANA dsDNA positive	Lupus nephritis

^a^Abbreviations: ANA, antinuclear antibody;ASo, antistreptococcal, ds DNA; double stranded deoxyribo nucleic acid; USG, ultrasonography

## 5. Discussion 

Detection of silent renal diseases by urinary screening test is one strategy to reduce the burden of CKD in the pediatric population. Dipstick urinalysis is the most common test used for detecting urinary abnormalities in asymptomatic children (15). In the first screening, 5.5% of the children were found to test positive, and on further testing in the second screening, 0.71% children were found to test positive. Repeat screenings were performed to eliminate false positives. False positivity maybe due to exercise, exposure to cold, prolonged recumbence, and contamination of urine samples with menstrual blood in females (15).

Bakr et al. (16) reported urinary abnormalities in 1.3% of Egyptian school children in their first screening and it persisted in 0.72% in their second screening. In a Malaysian study, screening of school children for proteinuria and hematuria showed that 1.9% of those screened had positive results but only 0.12% were found to test positive on further evaluation (17). Shajari et al.(15) found that 4.7% of children tested positive in their first screening and only 1.4% in their second screening. However, a lower prevalence of urinary abnormalities (3.56%) was reported in elementary school children in Japan (11).

In contrast, a higher prevalence (9.6–30.3%) has been reported in the first urinary screening by authors (14,18,19) from different geographic regions of the world. Variation in the detection rate of urinary abnormalities on screening in these studies may be due to varying ethnic backgrounds and the prevalence of renal diseases in these populations. In our study, the male to female ratio was 0.84:1 in the first screening. Other authors (13, 20) have also shown that urinary abnormalities were more common in girls than in boys. Lin et al. (21) found abnormalities in more males than females. However, Vehaskari et al. (22) found that the prevalence of abnormalities was not age or gender dependent. The difference in these findings maybe due to a variation in the gender of children enrolled in the studies. Race had no effect on the result, as there was no difference in urinary abnormalities between Mongolian and non-Mongolian children.

Among the clinical parameters studied, only diastolic blood pressure was abnormal in those children who had urinary abnormalities. This may be because some of them were hypertensive due to underlying renal pathology. Overall IH was more common (0.40%) than IP (0.22%) and CHP (0.09%). In some studies IH was more common than IP (11, 16), while in others the reverse was found (17,23,24). Further, it was observed that those children who were already screened and sent to the hospital had a much higher incidence of IH (46.4% –60.1%), IP (4.9% –26.4%), and CHP(13.5% –17.5%) (12,13,22).

Five children (50%) had features of glomerulonephritis in the present study. Murakami et al. (11) from Japan and Bakr et al. (16) from Egypt reported glomerulonephritis in 76.6% and 66.6% of their children with confirmed urinary abnormalities, respectively. Four cases had features of lupus nephritis with positive serology and one had acute post-streptococcal glomerulonephritis, which subsequently resolved. lin et al. (25) from Taiwan also reported that the most common etiology was lupus nephritis (31.6%) in their children. However, Park et al. (13) from Korea found IgA nephropathy and Chao et al. (12) found mesangioproliferative glomerulonephritis (21.9%) and IgA nephropathy (11.3%) as predominant etiologies. However, Bergstein et al. (26) reported that no cause was discovered in 274 out of 342 children with microscopic hematuria and the most common cause of the disease was hypercalciurea (16%) in their series. Similarly, Chander et al. (27) found that 52.1% of children who were found to have silent abnormal urinalysis had no definite diagnosis, but organic kidney diseases and hypercalciurea accounted for 14.9% and 14.4%, respectively.

The urinary screening of school children by dipstick is a non-invasive and feasible test for early detection of silent renal diseases (28). At present there is no clear consensus for developing countries on whether screening programs for CKD in children and adolescents should be undertaken. Mass urinary screening programs are well established in some Asian countries (Japan, Korea, and Taiwan), but this is not the case for North America and Europe because of concern about cost-effectiveness. Sekhar et al.(29) analyzed the cost-effectiveness of urinary screening programs, found them to be an ineffective procedure for primary care providers, and supported the recommendations of the American Academy of Pediatrics guidelines (30). A major question for pediatric nephrologists in developing countries is what strategy should be adopted that can detect silent renal diseases that may manifest later in life.

In conclusion, early detection and prevention is increasingly important in clinical practice to help overcome the burden of the financial resources required to create dialysis and transplant centers, which are simply not available at most centers in developing countries. Such screening programs could have a long-term impact in reducing the burden of end stage renal disease in children.
